# Pathway analysis following association study

**DOI:** 10.1186/1753-6561-5-S9-S18

**Published:** 2011-11-29

**Authors:** Julius S Ngwa, Alisa K Manning, Jonna L Grimsby, Chen Lu, Wei V Zhuang, Anita L DeStefano

**Affiliations:** 1 Department of Biostatistics, School of Public Health, Boston University, 715 Albany Street, Boston, MA 02118, USA; 2 General Medicine Division, Massachusetts General Hospital; and Harvard Medical School, 250 Longwood Avenue, Boston, MA 02115, USA; 3 Department of Neurology, Boston University School of Medicine, 72 East Concord Street Boston, MA 02118, USA

## Abstract

Genome-wide association studies often emphasize single-nucleotide polymorphisms with the smallest *p*-values with less attention given to single-nucleotide polymorphisms not ranked near the top. We suggest that gene pathways contain valuable information that can enable identification of additional associations. We used gene set information to identify disease-related pathways using three methods: gene set enrichment analysis (GSEA), empirical enrichment *p*-values, and Ingenuity pathway analysis (IPA). Association tests were performed for common single-nucleotide polymorphisms and aggregated rare variants with traits Q1 and Q4. These pathway methods were evaluated by type I error, power, and the ranking of the VEGF pathway, the gene set used in the simulation model. GSEA and IPA had high power for detecting the VEGF pathway for trait Q1 (91.2% and 93%, respectively). These two methods were conservative with deflated type I errors (0.0083 and 0.0072, respectively). The VEGF pathway ranked 1 or 2 in 123 of 200 replicates using IPA and ranked among the top 5 in 114 of 200 replicates for GSEA. The empirical enrichment method had lower power and higher type I error. Thus pathway analysis approaches may be useful in identifying biological pathways that influence disease outcomes.

## Background

Genome-wide association studies (GWAS) have had successes in identifying novel genes related to diseases. In these studies the focus is often placed on the most significant single-nucleotide polymorphisms (SNPs) that pass a stringent genome-wide significance threshold. Furthermore, the variability explained by genome-wide significant SNPs is often substantially less than the proportion of heritability estimated for the disease [1]. With a stringent threshold, variants that confer small disease risks are more likely to be missed among the hundreds of thousands of SNPs that are tested. Hence additional methods that exploit genetic information beyond single SNP association testing of common variants are needed. Rather than focusing on individual SNPs or genes, we consider gene sets that may improve power to identify disease-related candidate genes or pathways. The gene set enrichment analysis (GSEA) developed by Subramanian et al. [2] was one of the first approaches developed to identify gene sets that are associated with phenotypes of interest based on gene expression data. In another study, Mootha et al. [3] examined expression levels of 22,000 genes in a microarray study of diabetes and found that no gene showed a statistically significant expression difference after adjustment for multiple testing. However, by using a pathway-based approach, Mootha and colleagues were able to identify a set of PGC-1α responsive genes that showed a modest but consistent change in expression levels in muscle samples from subjects with diabetes [3]. Wang et al. [4] later demonstrated that pathway-based approaches, which jointly consider many contributing factors in the same pathway, might complement GWAS. Using results from GWAS, these investigators have used their approaches to test the association of pathways to determine whether a set of genes from a biological pathway is associated with a disease trait of interest, although the variants individually may not necessarily meet the genome-wide association threshold. These approaches have been useful and have provided plausible biological insights into underlying disease mechanisms.

In the present study, our main focus was to test the hypothesis that using gene set information in pathway analysis approaches will improve identification of disease-related genes or pathways. We implemented three pathway approaches: gene set enrichment analysis (GSEA), assessment of empirical enrichment of gene sets, and Ingenuity pathway analysis (IPA) (using the IPA software from Ingenuity Systems) [5]. The GSEA ranks genes by their most significant SNP and looks for gene sets with genes at the top of the ranked list. The empirical enrichment method seeks to find gene sets with larger than expected proportions of genes with association *p*-values less than 0.01. The IPA software identifies a set of focus genes, defined as genes containing SNPs that meet a user-specified level of nominal significance. The overall goal of our analysis is to compare these three approaches on the basis of their ability to identify pathways significantly associated with the outcome while maintaining a low type I error rate.

## Methods

The data analyzed in this study were from a mini-exome scan that used real sequence data for 3,205 genes donated by the 1000 Genomes Project; the data were made available by Genetic Analysis Workshop 17 (GAW17) [6]. A total of 24,487 SNPs were mapped to the exon-sequenced data spanning all autosomes in 697 unrelated individuals. Most of the SNPs were rare variants with more than half of the SNPs in the sample having a minor allele frequency (MAF) below 1% and with only a tenth having a MAF above 5%.

We analyzed two continuous traits, Q1 and Q4. These traits were simulated as normally distributed phenotypes. The simulating model for Q1 includes variants from nine genes in the VEGF pathway; hence the VEGF pathway is considered the truly associated pathway. The generating model for Q4 does not include any of the genotyped exonic SNPs. Consequently, any statistically significant associations with Q4 are considered false positives.

### Association analysis

Single-SNP linear association tests of common SNPs (MAF at least 1%) were performed assuming an additive model for SNP effects. Covariates included in the linear regression models were Age, Smoking, and Sex. Although Age and Sex were fixed, the smoking status covariate varied across the 200 replicates. To incorporate rare variants, the count of rare alleles (MAF < 1%) observed within a gene was computed for each gene and was used as an independent variable within a regression model [7,8]. We refer to this genetic variable as the aggregate rare variant. We used PLINK (http://pngu.mgh.harvard.edu/purcell/plink) [9] and R software (R Development Core Team; http://www.R-project.org) [10] to perform linear regressions for common and rare SNPs, respectively. The association tests were performed on all replicates of the simulated phenotype data.

Seven ethnic groups are represented in the sample. These ethnic groups are Centre d'Etude du Polymorphisme Humain (CEPH) (European-descent residents of Utah) (90), Denver Chinese (107), Han Chinese (109), Japanese (105), Luhya (108), Tuscans (66), and Yoruba (112) [6]. Population stratification (or structure) among these ethnic groups can cause spurious association. Thus we decided to adjust for population structure. Initially, we created dummy variables for each ethnicity and fitted them as covariates to the model. Examination of the QQ plots showed inflated genomic control value (*λ* > 2.0) for Q1 but not for Q4 when adjusting for ethnic group. We then implemented a principal components (PC) analysis, a popular method in GWAS for identifying and adjusting for subtle population structure; we used the PCs instead of the ethnicity dummy variables. Principal components were obtained for common SNPs. PC1, PC2, PC3, PC4, and PC9 were significantly associated (*p* < 0.05) with Q1. PC1, PC4, and PC6 were significantly associated with Q4. We reexamined the QQ plots after adjustment for the PCs significantly associated with each outcome. The genomic control value was substantially reduced for Q1, and hence we included significant PCs in the regression models.

The success of pathway analysis depends on the association tests of individual SNPs provided as input. A common approach for these methods is to perform the genome-wide association analysis, represent a given gene by one or more SNPs, feed the output results into the pathway-based methods, and later identify significant pathways. In the current study, we assigned each gene the *p*-value of the most significant genetic variable within that gene from association analysis, where the genetic variable was either a single common SNP or the aggregate rare variants from that gene.

### Pathway analysis

We implemented two gene set analyses: (1) the GSEA algorithm described by Wang et al. [4] and (2) a test of empirical enrichment similar to that described by Chasman [11]. For these two approaches, we used the curated gene sets from the Molecular Signature Database (MSigDB, version 2.5) [12]. These gene sets were drawn from online pathway databases, publications in PubMed, and the knowledge of domain experts. In total, 601 gene sets with between 10 and 409 genes were available for these analyses, with genes belonging to one or more gene sets. A third approach was the canonical pathway analysis available in IPA. All three methods were applied to each of the 200 replicate data sets.

### Gene set enrichment analysis

The GSEA is a nonparametric procedure that ranks all the genes by their *p*-value, which is obtained from the association analysis, as described earlier. Each gene is assigned a test statistic value *r_i_* obtained directly from its *p*-value. The genes are ranked from highest to lowest: *r*_1_, *r*_2_, …, *r_N_*. We compute an enrichment score for each gene set *S*:(1)

A total number of *N* genes are represented by all the SNPs. Each gene set *S* is composed of *N_H_* genes. The result of the algorithm is an enrichment score (ES). The ES(*S*) is a weighted Kolmogorov-Smirnov-like running sum statistic. It describes the overrepresentation of the *N_H_* genes at the top of the entire ranked list of genes. If the genes in gene set *S* are associated with the outcome and appear at the top of the ranked list of genes, the enrichment score tends to be high. A high ES indicates that the gene set has more genes with low *p*-values than would be expected by chance alone.

We followed the procedure suggested by Wang et al. [4] to compute a normalized enrichment score (NES) so that the NES can be compared across gene sets and the empirical *p*-values of the NES can be obtained. The NES is calculated by permuting the affection status of the sample 1,000 times, performing the association analysis with common SNPs and the aggregated rare variants and recomputing the ES for each gene set. The NES for a particular gene set is computed by normalizing the observed ES with the average and standard deviation (SD) of the ESs across the permutations. The permutations of the data sets are denoted by *π*:(2)

An empirical *p*-value can be obtained by comparing the observed NES to the null distribution of NES values from the permutations. Thus a gene set has a significant NES and is significantly enriched with genetic variant associations if the empirical *p*-value is less than the specified alpha level, which in this application was chosen to be a conservative value of 0.01.

### Empirical enrichment

The empirical enrichment analysis was used to determine whether gene sets had a higher proportion of genes with association *p*-values less than a significance level of 0.01 than would be expected by chance. A high proportion of low *p*-values would indicate that a gene set is enriched with genetic variant associations. Once again, the *p*-value for the gene was taken from the minimum association *p*-value from either the common SNPs or the aggregate rare variants. The proportion of genes with *p*-values less than 0.01 was recorded for each gene set and compared to a null distribution of the same proportion observed in the 1,000 permutations. A gene set was considered significantly enriched if the empirical *p*-value was less than 0.01.

### Ingenuity pathway analysis

In addition to the two pathway analyses described already, we implemented the IPA on the same set of association results. A set of focus genes was identified in IPA as those genes with an association *p*-value less than 0.01 (either a single SNP within the gene or the aggregate rare variants of the gene). Canonical pathways analysis identified the pathways from the IPA library of canonical pathways that were most significant given the focus genes. The significance of the association between the focus genes and the canonical pathway was measured in two ways: (1) as the ratio of the number of molecules from the focus gene set that map to the pathway to the total number of molecules that map to the canonical pathway and (2) using Fisher’s exact test, which was used to calculate a *p*-value to determine the probability that the association between the focus genes and the canonical pathway is explained by chance alone.

### Type I error and power

All three methods of pathway analysis were evaluated for power and type I error. A gene set was considered statistically significant if the *p*-value obtained by the analysis method was less than a predetermined alpha level of 0.01. GSEA produces an empirical *p*-value of the normalized enrichment score, the empirical enrichment produces an empirical *p*-value of the number of genes in the gene set with *p*-values less than 0.01, and IPA produces a nonparametric *p*-value from Fisher’s exact test.

To determine power, we tested the null hypothesis that the VEGF pathway is not associated with the trait versus the alternative hypothesis that the VEGF pathway is associated with the trait in each replicate. Given that the Q1 trait was simulated from nine genes in the VEGF pathway, the VEGF pathway was considered the truly associated pathway. The estimate of power is the proportion of replicates in which the pathway methods detect a significant enrichment of the VEGF pathway.

The Q4 trait was simulated independent of any of the genetic variants distributed. Consequently, any significant associations with Q4 can be considered false positives, and we used this trait to evaluate type I error. For each replicate, the type I error is estimated by the proportion of gene sets in which the pathway methods detect significant enrichment.

## Results

### Association analysis

The analysis of variance (ANOVA) for the quantitative trait Q1, by ethnicity, showed no significant difference in means among the seven ethnic groups for Q1 (*F* = 1.62, df = 6, *p* = 0.1387). There was a significant difference in means for Q4 by ethnicity (*F* = 11.39, df = 6, and *p* < 0.0001). The genomic control value (*λ*), which is based on the genome-wide association of common variants with no ethnicity adjustment, a dummy variable adjustment for ethnicity, and a PC adjustment, was computed using replicates 1 and 100 (Table 1). The inflated *λ* observed for Q1 was not totally mitigated by adjusting for ethnicity using dummy variables. PC adjustment substantially reduced *λ* for Q1. Based on these findings, for all subsequent analyses we chose to adjust for the PCs associated with Q1 and Q4.

**Table 1:** Genomic control (*λ*) based on association testing of all common variants (MAF ≥ 0.01)

Trait	No ethnicity adjustment		Ethnicity (dummy variables)		Principal components	
	
	Replicate 1	Replicate 100	Replicate 1	Replicate 100	Replicate 1	Replicate 100
Q1	2.525	2.654	1.649	1.639	1.040	1.086
Q4	0.915	0.916	1.011	0.964	0.966	0.929

All models were adjusted for age, sex, and smoking with columns representing different ethnicity adjustments.

The association analysis of the Q1 trait with single common SNPs and the rare variant aggregate for each gene identified a minimum of 94 and a maximum of 271 genetic variants with *p*-value less than 0.01 among all of the 200 replicates. The number of significantly associated genetic variants identified for the Q4 trait ranged between 43 and 229. SNPs C13S523 and C13S522 were the most significant SNPs among all the replicates, with median *p*-values of 2.4 × 10^−13^ and 1.215 × 10^−9^, respectively. These two SNPs were from the *FLT1* gene, one of the nine simulated genes for the VEGF pathway. Across all 200 replicates only *FLT1* yielded a genome-wide significant median *p*-value among the genes in the VEGF pathway (Table 2). *FLT1* was ranked as the top gene among all the replicates. Within the VEGF pathway the next most strongly associated SNP was KDR with a median ranking of 34 and a median *p*-value of 0.0017.

**Table 2:** Rank and nominal *p*-value from the association test between Q1 and the genes in the VEGF pathway included in the simulating model for Q1

	*ARNT*	*ELAVL4*	*FLT1*	*FLT4*	*HIF1A*	*HIF3A*	*KDR*	*VEGFA*	*VEGFC*
Rank	112	1,007	1	1,013	698	544	34	985	214
*p*-value	0.009	0.1359	2.4 × 10^−13^	0.1441	0.0885	0.0616	0.0017	0.1380	0.0186

The smallest *p*-value from either single SNP association of common variants or aggregate of rare variants within a gene was chosen to represent the gene. All models were adjusted for age, sex, smoking, and principal components.

### Type I error and power

**Figure 1:**
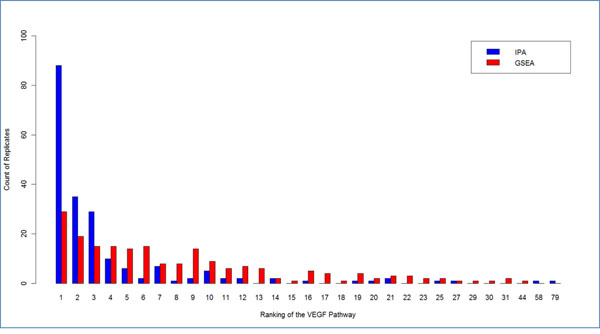
Histogram, across replicates, of the ranking of the VEGF pathway among all candidate pathways using Ingenuity pathway analysis and gene set enrichment analysis.

Type I error and power were computed to assess the performance of the three methods (Table 3). For the power calculations we focused on the VEGF pathway. Both GSEA and IPA had high power for detecting the VEGF pathway for the Q1 trait (91.2% and 93%, respectively). The VEGF pathway was ranked 1 or 2 in 123 out of 200 replicates by IPA and 48 out of 200 replicates by GSEA (see Figure 1). IPA performed the best to detect the pathway under which the data set was simulated. The empirical enrichment method had a lower power (42.9%) compared to the other two methods. The median type I error is reported in Table 3. GSEA and IPA were more conservative, with lower type I error compared to the empirical enrichment method. The type I error for the empirical enrichment method was close to the nominal level.

**Table 3:** Comparison of type I error and power calculations among all three methods

Method	Power	Median type I error
GSEA	0.912	0.0083
Empirical enrichment	0.429	0.0133
IPA	0.930	0.0072

## Discussion and conclusions

We used gene set information and pathway analysis approaches to identify disease-related candidate genes or pathways using three methods. Rather than focusing on individual SNPs or genes, we considered pathways containing genes sets that together may improve power to identify disease-related candidate genes or pathways. We assessed the performance of these three methods by computing the power and type I error rate. Both the GSEA and IPA approaches had high power and deflated type I error rate. The GSEA and IPA pathway methods identified the primary gene set under which the data set was simulated (VEGF pathway) for the Q1 trait. IPA performed the best to detect this pathway, which may not be surprising given that the VEGF pathway as defined within IPA was used in the simulation model. The VEGF gene set used for the implementation of GSEA in this study was not exactly the same as the VEGF pathway under which the data were simulated, yet this method still had high power to detect this pathway. The empirical enrichment approach had a lower power compared to the other methods.

We observed conservative type I error for the pathway approaches implemented in the current study. However, the number of significant findings will depend on the number of genes being considered and the number of pathways. For IPA the number of significant findings will also depend on the number of focus genes. The mini-exome data contained several thousand genes; however, in full genome-wide data the number of genes under consideration would be an order of magnitude greater. In IPA a Benjamini-Hochberg multiple comparison adjustment can be applied and may be appropriate when searching across all pathways using association results from genome-wide data, but we did not apply this adjustment in the current study. We consider the GSEA and empirical enrichment methods to be hypothesis-generating methods, useful for highlighting SNPs or genes that may be of biological significance. We used an alpha level of 0.01 rather than a Bonferroni-corrected alpha level for the number of gene sets tested. The empirical *p*-values do control for the correlation between the gene sets, so such a correction would be appropriate if the goal of the study was different.

Association analysis detected only a single gene in the VEGF pathway at a genome-wide significance level. Using a more liberal threshold, such as *p* < 0.01 results in the identification of many genes (hundreds of genes for some replicates) associated with Q1. Methods that enable researchers to identify true associations among these interesting but not genome-wide significant genes are required. The current study shows the value of pathway analysis; GSEA and IPA were able to correctly identify the VEGF pathway as important for the Q1 trait. Identification of this pathway helps to narrow the focus among the interesting hits to genes within the VEGF pathway and provides biological insight into the Q1 trait.

The success of pathway approaches depends on the association analysis results used as input. In the current study we performed single SNP association testing of common variants and of counts of rare variants within a gene. We observed that association analysis of only common SNPs missed several genes in the generating model (results not shown). For the Q1 trait there were causative genes that did not have any common SNPs associated at the 0.01 level, indicating a potential limitation of studying only common SNPs. Even when we included the aggregate rare variants in the association analysis, we did not detect all the genes associated in the VEGF pathway at a genome-wide significance level. Other methods of association analysis that use common and rare variants may further improve the power of pathway analysis by refining the input to these methods.

One limitation of GSEA is that overrepresentation of genes with nominally significant associations from within a specific pathway may not be driven by true association. If genes with high levels of linkage disequilibrium between them are in the same gene set, then that gene set may appear to be enriched, which in the case of false-positive association will lead to inflated type I error. Another shortcoming of GSEA is that if implemented in-house, it requires manual updating by the investigator to maintain current gene sets. Such manual updating is not required when using the IPA software. However, there is no cost associated with GSEA scripts compared to the commercial IPA product.

The failure of SNPs identified as genome-significant in GWAS to explain the heritability of the phenotype underscores the importance of looking beyond the most significant SNPs or genes and searching for additional variants with moderate statistical significance. Our study suggests that the IPA and GSEA methods can aid in the detection of variants with moderate significance. In conclusion, using gene set information and pathway analysis approaches may yield useful information in genetic association studies of human diseases.

## Competing interests

The authors declare that there are no competing interests.

## Authors’ contributions

JSN, AKM, WVZ and ALD conceived this study. The data were cleaned by JSN and AKM. Association analyses were performed by JSN, AKM, WVZ and CL. The empirical enrichment analyses were performed by AKM. The Ingenuity Pathway Analysis was done by JLG and ALD. The manuscript was written by JSN. All authors contributed toward reviewing and revising the final manuscript.
